# Cropping Systems and Cultural Practices Determine the *Rhizoctonia* Anastomosis Groups Associated with *Brassica* spp. in Vietnam

**DOI:** 10.1371/journal.pone.0111750

**Published:** 2014-11-05

**Authors:** Gia Khuong Hoang Hua, Lien Bertier, Saman Soltaninejad, Monica Höfte

**Affiliations:** Laboratory of Phytopathology, Department of Crop Protection, Faculty of Bioscience Engineering, Ghent University, Gent, Belgium; Agriculture and Agri-Food Canada, Canada

## Abstract

Ninety seven *Rhizoctonia* isolates were collected from different *Brassica* species with typical *Rhizoctonia* symptoms in different provinces of Vietnam. The isolates were identified using staining of nuclei and sequencing of the rDNA-ITS barcoding gene. The majority of the isolates were multinucleate *R. solani* and four isolates were binucleate *Rhizoctonia* belonging to anastomosis groups (AGs) AG-A and a new subgroup of A-F that we introduce here as AG-Fc on the basis of differences in rDNA-ITS sequence. The most prevalent multinucleate AG was AG 1-IA (45.4% of isolates), followed by AG 1-ID (17.5%), AG 1-IB (13.4%), AG 4-HGI (12.4%), AG 2-2 (5.2%), AG 7 (1.0%) and an unknown AG related to AG 1-IA and AG 1-IE that we introduce here as AG 1-IG (1.0%) on the basis of differences in rDNA-ITS sequence. AG 1-IA and AG 1-ID have not been reported before on *Brassica* spp. Pathogenicity tests revealed that isolates from all AGs, except AG-A, induced symptoms on detached leaves of several cabbage species. In *in vitro* tests on white cabbage and Chinese cabbage, both hosts were severely infected by AG 1-IB, AG 2-2, AG 4-HGI, AG 1-IG and AG-Fc isolates, while under greenhouse conditions, only AG 4-HGI, AG 2-2 and AG-Fc isolates could cause severe disease symptoms. The occurrence of the different AGs seems to be correlated with the cropping systems and cultural practices in different sampling areas suggesting that agricultural practices determine the AGs associated with *Brassica* plants in Vietnam.

## Introduction

**Figure 1:**
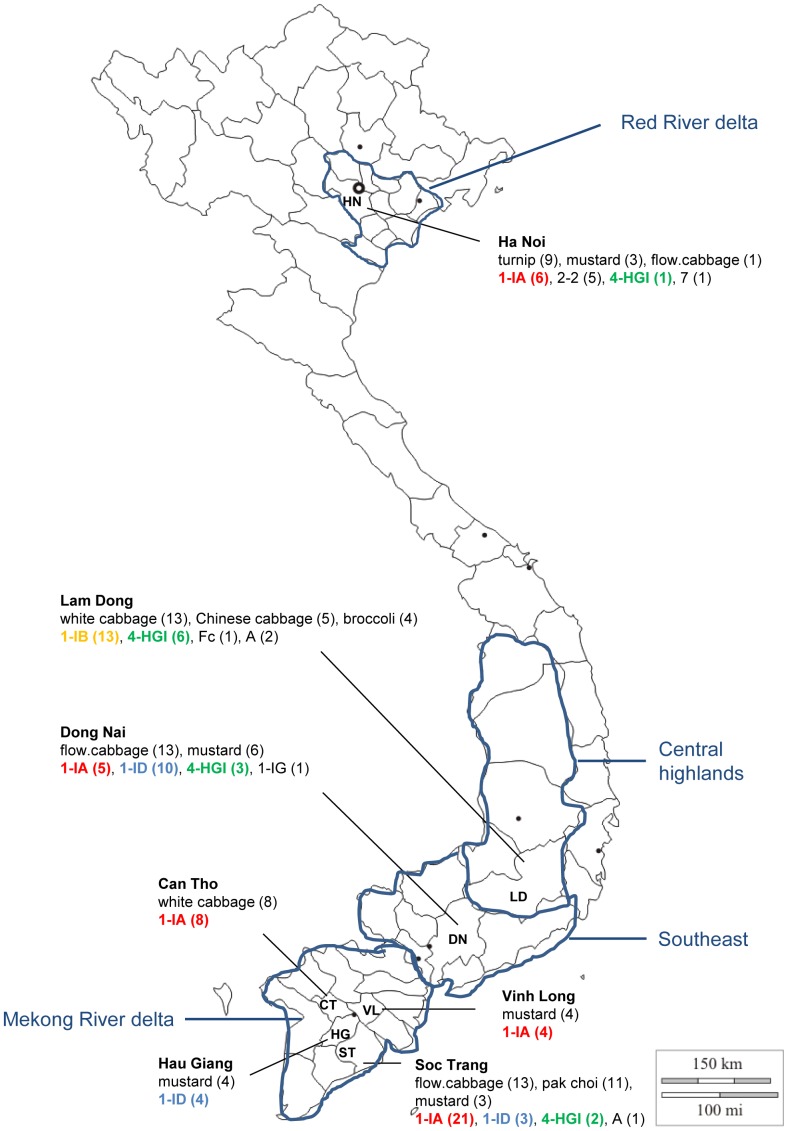
Location of sites for collection of *Rhizoctonia* isolates from *Brassica* spp. in Vietnam. The seven provinces sampled are: Ha Noi (districts of Gia Lam, Thanh Tri and Dong Anh), Lam Dong (Da Lat city and Duc Trong district), Dong Nai (Bien Hoa city), Vinh Long (Binh Tan district), Can Tho (Cai Rang district), Hau Giang (Phung Hiep district) and Soc Trang (Soc Trang city and My Xuyen district). In each city or district of one province, one to two wards were surveyed and these wards are marked with a start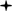. Different colors are used to highlight the most important AGs found in our survey including AG 1-IA, AG 1-IB, AG 1-ID and AG 4-HGI.

Vietnam is a country in Southeast Asia in which the agricultural sector accounts for more than 22% of the GDP, 30% of export and 52% of all employment. Vietnam is not only one of the world leaders in rice and coffee export, but also the third world's largest vegetable producer. *Brassicas* are among the main vegetables produced for both local consumption and export [1]. Vegetables in Vietnam are mainly produced by poor households living in the Red River and Mekong River delta (see Figure 1) in intensive cultivation systems or in rotation with other crops. Due to the lack of knowledge in crop management, limited availability of technology and land fragmentation, farmers are suffering heavy yield losses year after year. Among the limiting factors in vegetable production is the occurrence of *Rhizoctonia* diseases, which has been recognized as one of the most important threats.

*Rhizoctonia* is a genus of basidiomycete fungi causing many important plant diseases. Based on differences in the number of nuclei per cell, *Rhizoctonia* isolates have been differentiated into uninucleate *Rhizoctonia*, binucleate *Rhizoctonia* (teleomorphs: *Ceratobasidium* spp. and *Tulasnella* spp.) and multinucleate *Rhizoctonia* (teleomorphs: *Thanatephorus* spp. and *Waitea* spp.) [2]. *Rhizoctonia* species can also be classified using biochemical and molecular techniques. Among those, rDNA-ITS sequence analysis appears to be the most convenient and reliable method [3]. Currently, isolates of *R. solani*, the most widely recognized species within the multinucleate *Rhizoctonia* group, have been divided into 13 anastomosis groups (AGs), while 16 AGs of binucleate *Rhizoctonia* have been recognized [2]–[7]. Due to the considerable genetic diversity, several AGs have been further divided into subgroups based on phylogenetic differences. These phylogenetic differences can be associated with differences in morphology, ecology, pathogenicity and biochemical characteristics, although this is not necessarily the case [8].

Compared to binucleate *Rhizoctonia*, multinucleate *R. solani* AGs usually have a wider host range and higher virulence. *R. solani* can survive for a long period in plant debris, contaminated seeds, or infested soils as mycelium or sclerotia [9], [10]. Under favorable conditions, sclerotia geminate and form delicate hyphae that will grow toward the host plants [11]. *Brassica* vegetables can be attacked by several different AGs of *R. solani* resulting in the development of various diseases such as foliar blight, wirestem and damping off. In previous studies, AGs 1-IB, 1-IC, 2-1, 2-2 IIIB, 3, 4-HGI, 4-HGII, 4-HGIII, 5, 7, 9 and 10 were shown to be pathogenic on *Brassica* crops grown in Canada [12], [13], Australia [14], Japan [15], [16], North America [17]–[19], Brazil [20], China [21], Belgium [22], and the UK [23]. Although *Rhizoctonia* diseases occur severely and frequently on leafy vegetables cultivated in Vietnam, there have been no reports about the AGs and subgroups that attack *Brassica* crops. Therefore, our research aimed at (i) identifying the species and AGs of *Rhizoctonia* present on *Brassica* plants in different vegetable producing regions in Vietnam, and (ii) verifying the susceptibility of *Brassica* vegetables to *Rhizoctonia* isolates collected. Our story revealed that *Rhizoctonia* AGs such as AG 1-IA and AG 1-ID, which have not been reported before on *Brassica* spp., are predominant in Vietnam, which is presumably linked with the cultural practices and cropping systems in the different sampling areas. Moreover, we describe two new AGs that were previously unknown.

## Materials and Methods

### Field sampling and pathogen isolation

Sampling was done on private farms by Gia Khuong Hoang Hua (Vietnamese citizen) with permission from the farmer. In general, permission by the authorities was not required since the sampling studies were carried out on private farms. Only cabbage plants showing symptoms of the undesired *Rhizoctonia* fungus were sampled, hence the field studies did not involve endangered or protected species. The GPS coordinates of the locations where samples were collected are presented in Table S1 in File S1.

The survey was conducted from September to October 2011 on various fields in the Red River delta (Ha Noi; an important vegetable production area of the North), the Mekong River delta (Vinh Long, Can Tho, Hau Giang and Soc Trang; main vegetable production areas of the South), the Central Highlands (Lam Dong; vegetables are mainly produced for export) and the Southeast (Dong Nai; vegetables are mainly produced for domestic consumption) (Figure 1). These regions were chosen because of their importance in vegetable production and because they offer a good representation of the different climatic and topographic conditions in Vietnam and, therefore, a good representation of the distribution of *Rhizoctonia* spp. on *Brassica* spp. in Vietnam can be obtained. Total production area, climatic conditions and main agricultural activities of these regions are listed in Table 1.

**Table 1:** Sampling locations and their relevant characteristics [53]–[59].

Sampling location	Agricultural area (1000 ha)	Average temperature (°C)	Main crop
**Red River Delta**			
Ha Noi	152.24	24	- Cabbage, tomato, cucumber, radish
			- Rice
**Central highlands region**			
Lam Dong	279.00	High land: 14	- Lettuce, cabbage, carrot, potato
		Low land: 21	- Coffee, tea, cashew-nut tree, cotton
**Southeast region**			
Dong Nai	289.02	27	- Coffee, cotton, black pepper
			- Durian, grapefruit, mango
**Mekong River Delta**			
Vinh Long	116.18	27	- Rice
			- Mango, orange, grapefruit, durian
			- Cabbage, cucumber, bean
Can Tho	115.00	27	- Rice
			- Cabbage, cucumber
Hau Giang	139.07	27	- Durian, pineapple, grapefruit
			- Rice
Soc Trang	278.15	27	- Rice
			- Grapefruit, mango, durian

A total of 142 *Brassica* plants with *Rhizoctonia*-like symptoms were sampled. Infected root and leaf tissues were washed in running tap water, surface-disinfected in 1% sodium hypochlorite solution for two min and then rinsed twice in sterile water before placing on 1% water agar medium supplemented with streptomycin (0.05 g L^−1^). After 24 h of incubation, *Rhizoctonia*-like hyphal tips growing out of these tissues were transferred to fresh potato dextrose agar (PDA; Difco) plates and incubated for two to four days at 28°C.

Ninety seven *Rhizoctonia* isolates were recovered and subjected to nuclei staining and sequencing of the ITS-rDNA region. All isolates are listed in Table 2.

**Table 2:** Characterization of *Rhizoctonia* isolates collected from diseased *Brassica* crops grown in Vietnam by sequencing the ITS-region.

AG/Subgroup	Host plant	Isolate^ab^	Genbank accession numbers
1-IA	*B. parachinensis* (Chinese flowering cabbage)	STST03-1, STST03-3, STST03-4, STST04-2, **STMX04-1, STMX04-2, STMX04-3, STMX04-4**, STMX04-5	KF907702, KF907703, KF907704, KF907705
	*B. juncea* (Mustard cabbage)	DNBH01-1, DNBH01-2, DNBH01-3, DNBH02-2, DNBH02-3	
		HNGL01-1, HNGL01-2, **HNGL01-3**	KF907706
		STST02-1, STST02-2	
		VLBT01-1, VLBT01-2, VLBT01-3, VLBT01-4	
	*B. chinensis* (Pak choi)	**STMX01-1**, STMX01-2, **STMX01-4**, STMX01-5, STMX02-1, STMX02-2, **STMX02-3**, **STMX03-1**, **STMX03-2**, STMX03-3	KF907707, KF907708, KF907709, KF907710, KF907711
	*B. oleraceae* (Turnip cabbage)	HNDD01-1, HNDD01-2, **HNDD01-3**	KF907712
	*B. oleraceae* (White cabbage)	CTCR01-1, CTCR01-2, **CTCR01-3**, **CTCR02-1**, **CTCR02-2**, CTCR02-3, CTCR03-1, CTCR03-2	KF907713, KF907714, KF907715
1-IB	*B. chinensis* (Chinese cabbage)	LDDT03-1	
	*B. oleraceae* (Broccoli)	LDDL01-1, **LDDL01-2**, LDDL01-3, LDDL01-4	KF907716
	*B. oleraceae* (White cabbage)	**LDDL04-1**, LDDL04-2, LDDL04-3, LDDL04-4, LDDL04-5, **LDDL05-1**, LDDL05-2, **LDDL05-3**	KF907717, KF907718, KF907719
1-ID	*B. parachinensis* (Chinese flowering cabbage)	**DNBH03-1**, **DNBH03-2**, **DNBH03-3**, DNBH03-4, DNBH03-5, DNBH05-1-1, DNBH05-1-3, DNBH05-2-2, **DNBH05-3-1**, **DNBH05-4**	KF907720, KF907721, KF907722, KF907723, KF907724
		STST04-1, STST04-3	
	*B. juncea* (Mustard cabbage)	HGPH01-1, HGPH01-2, **HGPH01-3**, HGPH01-4	KF907725
		STST02-3	
1-IG	*B. parachinensis* (Chinese flowering cabbage)	**DNBH05-1-2**	KF907730
2-2	*B. parachinensis* (Chinese flowering cabbage)	**HNTT01-1**	KF907726
	*B. oleraceae* (Turnip cabbage)	**HNDA01-1**, HNDA01-2, **HNDA01-3**, **HNDA01-4**	KF907727, KF907728, KF907729
4-HGI	*B. chinensis* (Chinese cabbage)	LDDT01-1, LDDT01-2, LDDL02-2	KF907731
	*B. parachinensis* (Chinese flowering cabbage)	DNBH05-2-1, DNBH05-3-2	
		STST01-1, **STST03-2**	KF907732
	*B. juncea* (Mustard cabbage)	DNBH02-1	
	*B. oleraceae* (Turnip cabbage)	**HNDD01-4**	KF907733
	*B. oleraceae* (White cabbage)	LDDT02-1, LDDT02-2, LDDT02-3	
7	*B. oleraceae* (Turnip cabbage)	**HNDA02-1**	KF907734
A	*B. chinensis* (Pak choi)	STMX01-3	
	*B. oleraceae* (White cabbage)	**LDDL03-1**, LDDL03-2	KF907735
Fc	*B. chinensis* (Chinese cabbage)	**LDDL02-1**	KF907736

a The first two letters represent provinces in which the samples were collected (i.e. CT: Can Tho, VL: Vinh Long, HG: Hau Giang, ST: Soc Trang, DN: Dong Nai, LD: Lam Dong and HN: Ha Noi).

b Isolates in bold (unique sequences) are submitted to Genbank.

### Nuclei staining

*Rhizoctonia* isolates were cultured on sterile glass slides covered by PDA for two days at 28°C. Actively growing fungal hyphae were stained with 10 µg mL^−1^ 4, 6-diamino-2-phenyl indole (DAPI; Sigma-Aldrich) and the number of nuclei per hyphal cell was determined using an Olympus BX51 microscope [22].

### DNA extraction, PCR and sequencing of the rDNA-ITS region

The rDNA-ITS region of all collected isolates was sequenced for identification to the AG and subgroup level. The usefulness of the rDNA-ITS region for identification of unknown *Rhizoctonia* spp. has been clearly shown by Sharon et al. [2], [3].

*Rhizoctonia* isolates were grown on potato dextrose broth at 28°C for one week. Mycelial mats were harvested by filtration and ground in liquid nitrogen to produce a fine powder. Total genomic DNA was extracted using the DNeasy Plant Mini Kit (Qiagen). The rDNA-ITS fragment including the 5.8 S gene was amplified using primers ITS4 (5′-TCCTCCGCTTATTGATATGC-3′) and ITS5 (5′-GGAAGTAAAAGTCGTAACAAGG-3′) [24]. The PCR amplification reactions were performed by adding 2 µL genomic DNA (5–10 ng µL^−1^) to 23 µL of reaction mixture containing 2.5 µL PCR buffer (10×; Qiagen), 5 µL Q-solution (Qiagen), 0.5 µL dNTPs (10 mM; Fermentas GmbH), 1.75 µL of each primer (10 µM), 0.15 µL Taq DNA polymerase (5 units µL^−1^; Fermentas GmbH) and 11.35 µL ultrapure sterile water. Amplification was performed using a Flexcycler PCR Thermal Cycler (Analytik Jena) programmed for an initial denaturation step at 94°C for 10 min followed by 35 cycles at 94°C for 1 min, 55°C for 1 min and 72°C for 1 min. Cycling ended with a final extension step at 72°C for 10 min. Amplification products were separated in 1% agarose gels in TAE-buffer at 100 V for 30 min and visualized by ethidium bromide staining on a UV transilluminator. The sequences of both strands were determined by LGC Genomics GmbH (Berlin, Germany) using Sanger sequencing.

### Identification using BLAST and phylogenetic analysis

Consensus sequences for all 97 isolates were created with BioEdit version 7.1.11. To determine the AG of the isolates, the rDNA-ITS consensus sequences obtained were compared to those in Genbank using the BLASTn tool.

However, since Genbank is an uncurated database, it can contain inaccurately designated *Rhizoctonia* spp., as has been previously shown by Sharon et al. [3]. Therefore, comparison of rDNA-ITS sequences of unknown isolates to a curated database of sequences containing representative rDNA-ITS sequences of all known uninucleate, binucleate and multinucleate *Rhizoctonia* AG and subgroups provides a more reliable identification. Such a database of representative sequences is available from Sharon et al. [2] and was provided by Michal Sharon to us. However, not all known AGs were present in this database, therefore we added representative isolates of the following AGs: AG 1-1E, AG 1-1F, AG 2-2 WB [25] and AG 13 [4]. The total number of sequences in the database was 129 and the Genbank accession numbers of all these sequences can be found in Table S2 in File S2.

Multiple alignments for multinucleate and binucleate *Rhizoctonia* isolates were constructed using MUSCLE which is implemented in MEGA 6 [26] and checked manually afterwards. The resulting alignments had a length of 717 bp (multinucleate *Rhizoctonia* isolates) and 768 bp (binucleate *Rhizoctonia* isolates).

Separate phylogenetic trees were constructed for multinucleate *Rhizoctonia* and binucleate *Rhizoctonia* isolates. For the binucleate tree, one representative isolate for each known binucleate AG was included together with the binucleate *Rhizoctonia* isolates obtained in this work.

For the multinucleate tree, reference isolates from the curated database (Table S2 in File S2) were added for all AGs present in our collection from Vietnam. Another 32 isolates from a characterization study in Vietnam that has not been published (Thuan et al., unpublished; Genbank accession numbers with prefix ‘EF’ in Table 3), and found on a range of crops and belonging to AGs AG 1-IA, AG 1-ID and AG 4-HGI were also added to the multinucleate *Rhizoctonia* alignment for phylogenetic analysis. Phylogenetic trees were built using the neighbour joining algorithm with 1000 bootstrap repeats using MEGA 6 [26]. Model testing was done using the software implemented in MEGA 6 and the K2+ G DNA substitution model was chosen.

**Table 3:** Multinucleate and binucleate *Rhizoctonia* isolates derived from Genbank included in the phylogenetic analysis for comparison.

AG/Subgroup	Isolate	Host plant	Origin	Genbank accession number	Reference
1-IA	L31-1, L66-1, L73, L59, L38, L52, L62-1	Rice	Vietnam	EF206342, EF429208, EF429211, EF429212, EF429210, EF429209, EF429207	unpublished
	RM61	Water spinach	Vietnam	EF429216	unpublished
	LB71	Water hyacinth	Vietnam	EF429215	unpublished
	DP38	Peanut	Vietnam	EF429214	unpublished
	CLV72-2	Barnyard grass	Vietnam	EF429213	unpublished
	BV71-2, BV61-2, BV50-1	Cotton	Vietnam	EF429206, EF429205, EF206341	unpublished
	CC72	Bermuda grass	Vietnam	EF429204	unpublished
	B34-1	Corn	Vietnam	EF429203	unpublished
1-IG	RMPG28	Chickpea	India	JF701750	[29]
1-ID	BV62-1, BV61-6, BV61-5, BV61-4, BV61-1	Cotton	Vietnam	EF197803, EF197804, EF197802, EF197801, EF197800	unpublished
	SR61, SR650	Durian	Vietnam	EF197798, EF197797	[30]
	B61-1	Corn	Vietnam	EF197796	unpublished
	CCD61-1	Sugar beet	Vietnam	EF197799	unpublished
4-HGI	XL4	Cauliflower	Vietnam	EF203247	unpublished
	CB63, CB34-2	Cabbage	Vietnam	EF203251, EF203245	unpublished
	CP50-2	Coffee	Vietnam	EF203250	unpublished
	BV68-1, BV68-2	Cotton	Vietnam	EF203249, EF203248	unpublished
	KT63-1	Potato	Vietnam	EF203246	unpublished
Fc	BS-YT-06-5-14, YT, BS-J-06-6-3, DL-jiang-06-2-4, DL-YT-06-4-10, DL-YT-06-4-9, DL-YT-06-3-4	Taro, Ginger	China	HM623619, HM623631, HM623615, HM623622, HM623625, HM623624, HM623623	unpublished

### Aggressiveness of *Rhizoctonia* isolates towards detached leaves of cabbages, rice and water spinach

**Figure 2:**
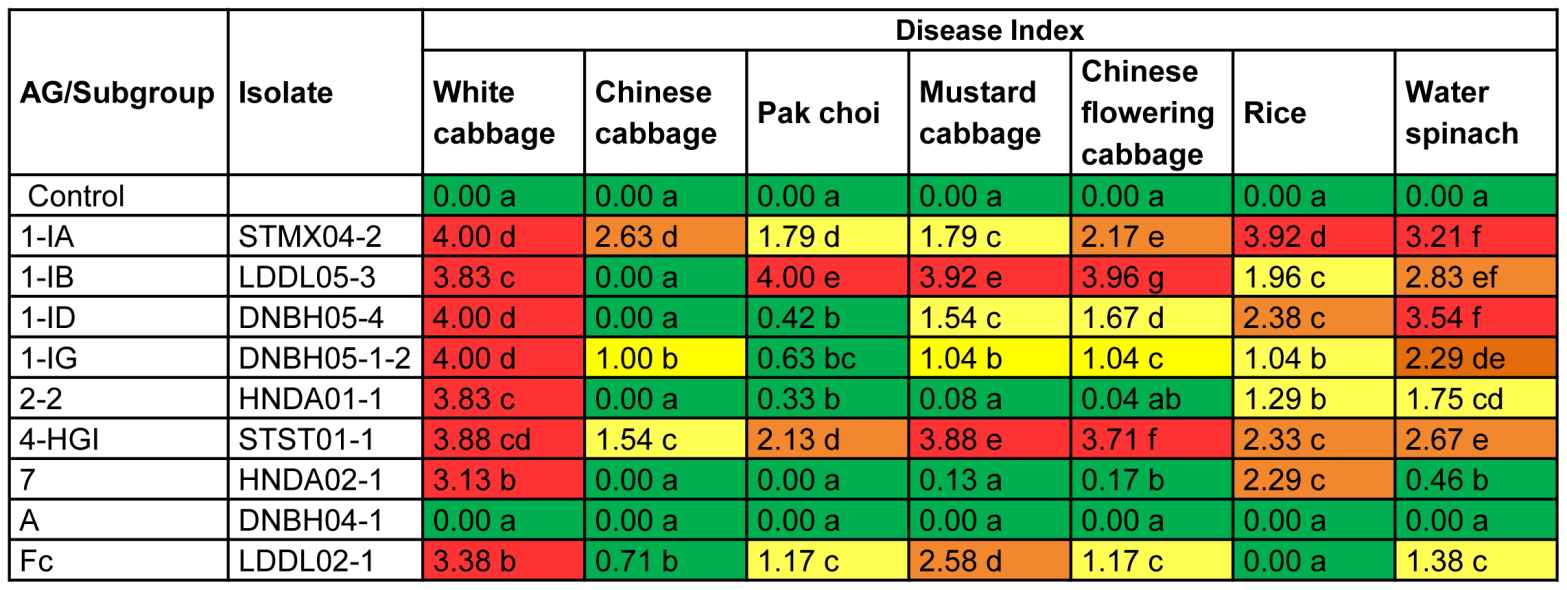
Aggressiveness of *Rhizoctonia* isolates towards detached leaves of white cabbage, Chinese cabbage, pak choi, mustard cabbage, Chinese flower cabbage, rice and water spinach. Leaves were scored using a scale ranging from 0 (no disease symptoms) to 4 (lesions covered more than 75% of leaf surface or dead leaf). For rapid visual evaluation of the data, a coloring scale with green (0<DI≤1), yellow (1<DI≤2), orange (2<DI≤3) and red (3<DI≤4) was used. The experiment was conducted twice and each treatment consisted of 12 leaves or leaf pieces. The data of the two experiments were pooled before Mann-Whitney comparisons were applied at p = 0.05. Within columns, disease severities followed by the same letter are not significantly different.

**Figure 3:**
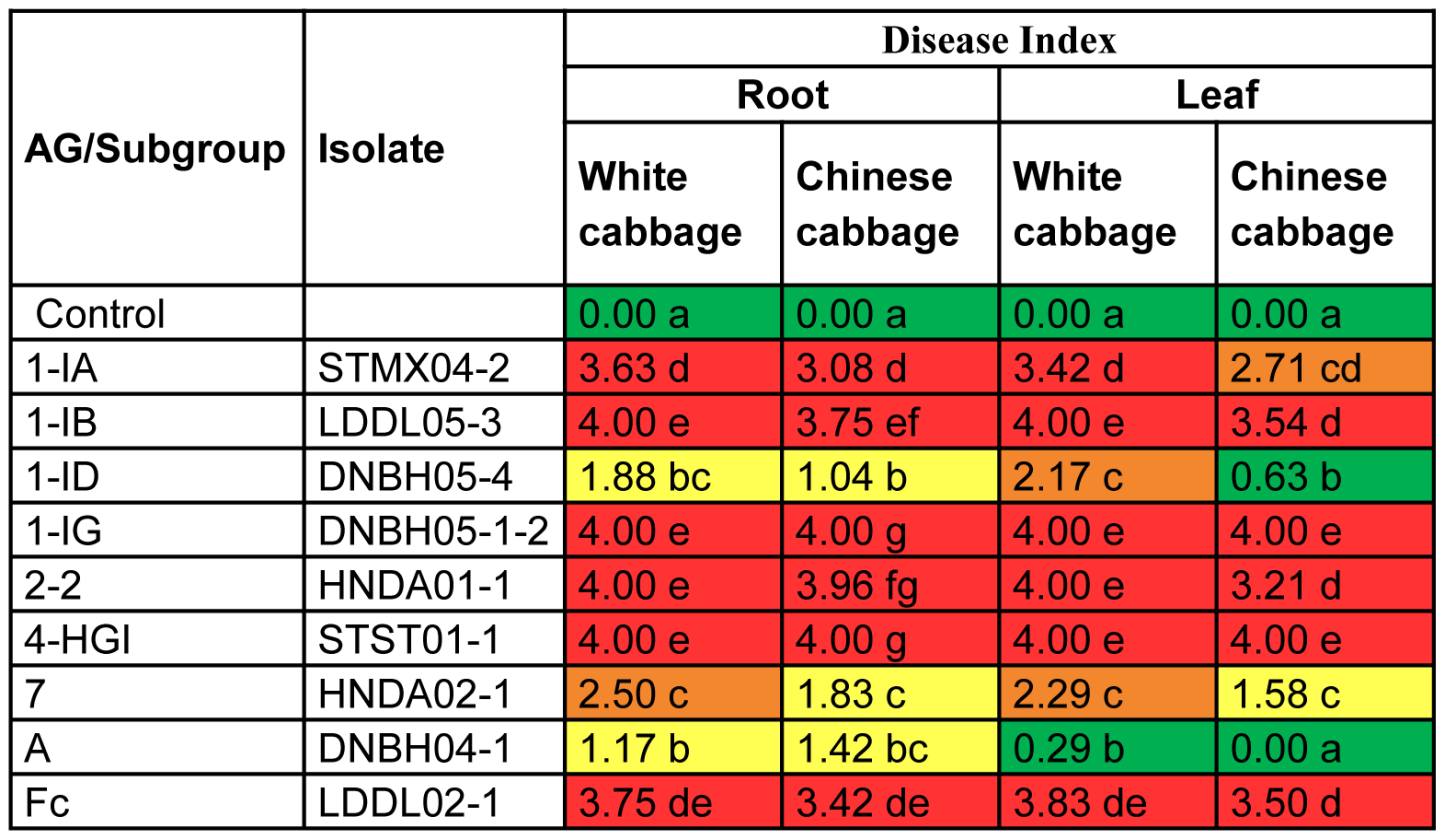
Pathogenic potential of *Rhizoctonia* isolates on seedlings of white cabbage and Chinese cabbage in *in vitro* bio-assays. Disease severity was assessed on a scale ranging from 0 (no symptoms) to 4 (lesions covering more than 75% of root, hypocotyl or leaf surface or dead plant). For rapid visual evaluation of the data, a coloring scale with green (0<DI≤1), yellow (1<DI≤2), orange (2<DI≤3) and red (3<DI≤4) was used. The experiment was done twice with 12 seedlings maintained in two square Petri plates for one treatment. The data of the two experiments were pooled before Mann-Whitney comparisons were applied at p = 0.05. Within columns, disease severities followed by the same letter are not significantly different.

**Figure 4:**
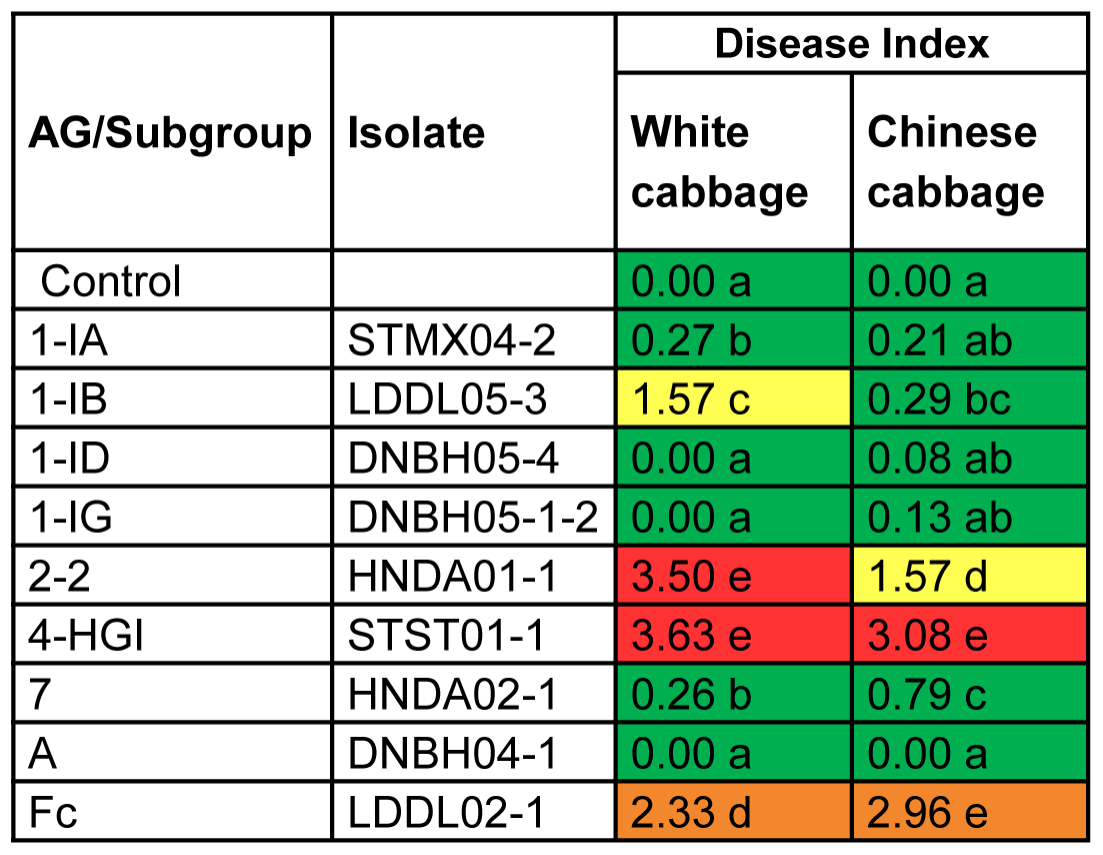
Pathogenic potential of *Rhizoctonia* isolates on roots and hypocotyls of white cabbage and Chinese cabbage in *in vivo* experiment. Disease severity on roots was assessed on a scale ranging from 0 (no symptoms) to 4 (seedling dead). For rapid visual evaluation of the data, a coloring scale with green (0<DI≤1), yellow (1<DI≤2), orange (2<DI≤3) and red (3<DI≤4) was used. The experiment was performed twice with 12 seedlings cultivated in two plastic boxes per treatment. The data of the two experiments were pooled before Mann-Whitney comparisons were applied at p = 0.05. Within columns, disease severities followed by the same letter are not significantly different.

Nine isolates (representing the nine different *Rhizoctonia* AGs collected) were randomly selected and tested for virulence towards several *Brassica* crops in two independent experiments (Figures 2, 3 and 4). To confirm their pathogenicity, tests were repeated for AGs 1-IA, 1-IB, 1-ID, 2-2, 4-HGI and A since each of these AGs consists of more than one isolate. Results of additional tests are shown in Tables S3, S4 and S5 in File S1.

Leaves of white cabbage (*Brassica oleracea*), Chinese cabbage (*B. chinensis*), pak choi (*B. chinensis*), mustard cabbage (*B. juncea*) and Chinese flowering cabbage (*B. parachinensis*) were cut into pieces (3×3 cm). The soil substrate used in our experiments was a mixture (w/w) of 50% potting soil (Structural; Snebbout, Kaprijke, Belgium) and 50% sand (Cobo garden; Belgium). Sets of six leaf discs were placed in a plastic box (16×11×6 cm) containing 400 g of soil substrate. Inoculum of *Rhizoctonia* spp. was produced according to the method described by Scholten et al. [27]. Briefly, water-soaked wheat kernels were autoclaved for 25 min on two successive days and then inoculated with three fungal discs (diameter 5 mm) cut at the edge of a 3-day-old *Rhizoctonia* colony cultured on PDA. Flasks containing the inoculated kernels were incubated for 14 days at 28°C and shaken every 3–4 days to avoid coagulation. Two *Rhizoctonia*-infected kernels which were comparable in size were buried 2 cm below each leaf disc. Leaf discs inoculated with sterile wheat kernels served as a control. All boxes were incubated in a growth chamber at 22°C.

The detached leaf bio-assay was also conducted to investigate the pathogenicity of the *Rhizoctonia* isolates on rice and water spinach, two important hosts of *Rhizoctonia* spp. in tropical countries. Surface-sterilized seeds of rice (*Oryza sativa* cv. CO39) and water spinach (*Ipomoea aquatic* cv. Trang Nong) were sown in plastic trays (45×45×10 cm) filled with 4 kg of soil substrate and kept in a growth chamber at 28°C for four weeks before their leaves were detached. Rice leaves were then cut into pieces (8 cm long) and six rice leaf pieces or six water spinach leaves were put in one square Petri dish containing a sterile filter paper moistened with sterile water. Two sterile glass slides were placed in the middle of each Petri dish to keep the leaves away from water. A 5-mm plug harvested from 3-day-old cultures of *Rhizoctonia* spp. on PDA was placed at the center of each leaf or leaf piece and the Petri dishes were incubated at 28°C.

After four days of incubation, disease severity was scored based on the following disease scale: 0 =  no symptoms observed; 1 =  lesions covered less than 25% of leaf surface; 2 =  lesions covered 25–50% of leaf surface; 3 =  lesions covered 50–75% of leaf surface; 4 =  lesions covered more than 75% of leaf surface or dead leaf. The experiment had a completely randomized design. Each treatment consisted of 12 leaves or leaf pieces equally divided into two boxes or Petri dishes.

### *In vitro* pathogenic potential of *Rhizoctonia* spp. on seedlings of white cabbage and Chinese cabbage

The same nine *Rhizoctonia* isolates were studied for their *in vitro* pathogenic potential using the method described by Keijer et al. [28]. Six surface-sterilized seeds of white cabbage (*B. oleracea* cv. TN180) or Chinese cabbage (*B. chinensis* cv. Elton) were germinated on Gamborg B5 medium (Gamborg B5 medium including vitamins; Duchefa) in a square Petri dish. Two mycelial disks (5 mm in diameter) from 3-day-old *Rhizoctonia* cultures grown on PDA were placed between seeds. In the control dishes, sterile PDA discs were used for inoculation. The Petri dishes were incubated at 22°C in the dark for two days for seed germination. Then, the second halves of the Petri dishes were covered with aluminum foil to protect the roots from light and placed in an upright position in a growth chamber (22°C, 12 h light). The disease severity was recorded for root and hypocotyl or for leaves after six days of incubation using the following disease scale: 0 =  healthy, no symptoms; 1 =  lesions covering less than 25% of the root, hypocotyl or leaf surface; 2 =  lesions covering between 25% and 50% of the root, hypocotyl or leaf surface; 3 =  wilted plant with lesions covering between 50% and 75% of the root, hypocotyl or leaf surface; 4 =  lesions covering more than 75% of root, hypocotyl or leaf surface or dead plant. In this test, a complete randomized design was applied with two Petri dishes (six seedlings each) per treatment and this experiment was done twice.

### *In vivo* pathogenic potential of *Rhizoctonia* spp. on roots and hypocotyls of white cabbage and Chinese cabbage

Surface-sterilized seeds of white cabbage and Chinese cabbage were germinated on wet filter paper in Petri dishes at 22°C one day before sowing into 600 g of soil substrate. Four days after sowing, each perforated plastic box (22×15×6 cm) with six seedlings was inoculated by placing a row of 12 *Rhizoctonia*-colonized wheat kernels in the middle of the box. The kernels used for inoculation had equivalent sizes and were produced as described previously. Control seedlings were similarly treated with sterile wheat kernels. All plants were incubated at 22°C. Disease severity on root and hypocotyl was evaluated 14 days after inoculation using the same disease scale described for the *in vitro* experiment. A completely randomized design was used with 12 seedlings cultivated in two experimental boxes per treatment and this test was performed twice with the same nine isolates that were used in the previous tests.

### Statistical analysis

The severity of *Rhizoctonia* diseases on roots and leaves of *Brassica* seedlings are presented in Figures 2–4 and Tables S3–S5 in File S1 as Disease index (DI). DI was calculated using the following formula:
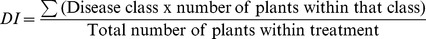



Pathogenicity data for the two experiments with nine isolates of nine different AGs were always very similar and no significant interaction was found between the experiments. Therefore, statistical analysis was done on pooled data for the different repeats. The non-parametric Kruskal-Wallis test for k independent samples was used, after which pair-wise comparisons were performed for all treatments using Mann-Whitney tests at a confidence level of p = 0.05.

To determine the correlation between the distributions of AGs and sampling locations and between the distributions of AGs and sampled *Brassica* spp., contingency tables were constructed using Excel. Then, potential significant differences between the variables were revealed with Fisher's Exact Tests. All statistical analyses were conducted in SPSS 22.0 (SPSSinc, Illinois, USA).

## Results

### Molecular characterization and phylogenetic analysis of *Rhizoctonia* isolates

A total of 142 *Brassica* plants with *Rhizoctonia*-like symptoms were sampled in various important vegetable producing regions in Vietnam (see Figure 1). Of the 97 *Rhizoctonia* isolates recovered, only four were binucleate with two nuclei per hyphal cell. The other 93 isolates had multinucleate cells (data not shown).

Analysis of the rDNA-ITS region using the BLASTn tool (against Genbank and against the curated database) revealed that three binucleate *Rhizoctonia* isolates belonged to AG-A while the fourth isolate (LDDL02-1) could not be assigned to any known AG.

Pairwise sequence similarity scores of isolate LDDL02-1 against all isolates in the curated database (representing all known AGs) were determined. Highest pairwise sequence similarities were found with isolates of multinucleate AG 6 (94%) and binucleate AG-Fb (93%) (Table 4). When blasting to Genbank, several isolates from taro and ginger from Yunnan Province in China were found to be nearly identical to isolate LDDL02-1.

**Table 4:** Pairwise sequence similarities of unknown isolates LDDL02-1 and DNBH05-1-2 to all known AGs from the curated database in Table S2 in File S2.

	LDDL02-1	DNBH05-1-2
**AG 1-IA**	0.88	0.92
**AG 1-IB**	0.84–0.87	0.84–0.87
**AG 1-IC**	0.89–0.90	0.89–0.90
**AG 1-ID**	0.85–0.86	0.85
**AG 1-IE**	0.89	0.94
**AG 1-IF**	0.84	0.85
**AG 2-1**	0.87–0.90	0.82–0.85
**AG 2-2**	0.86–0.87	0.84–0.85
**AG 2-3**	0.89–0.90	0.85
**AG 3**	0.89–0.90	0.84–0.85
**AG 4**	0.86–0.88	0.85–0.88
**AG 5**	0.91	0.86–0.87
**AG 6**	0.89–0.94	0.88–0.90
**AG 7**	0.91	0.89
**AG 8**	0.92–0.93	0.89–0.90
**AG 9**	0.9	0.86
**AG 10**	0.89–0.90	0.84–0.85
**AG 11**	0.88–0.89	0.84
**AG 12**	0.89–0.90	0.87–0.88
**AG 13**	0.90	0.90
**AG 2-BI**	0.85–0.86	0.81–0.82
**AG-A**	0.84	0.84
**AG-K**	0.84	0.84
**AG-Bb**	0.79–0.80	0.78–0.79
**AG-Q**	0.80	0.79
**AG-Bo**	0.83	0.83
**AG-Ba**	0.83–0.84	0.83–0.84
**AG-C**	0.80–0.82	0.82
**AG-H**	0.81–0.82	0.81
**AG-I**	0.81	0.80–0.81
**AG-D**	0.78–0.81	0.77–0.79
**AG-G**	0.85	0.85
**AG-L**	0.85	0.85–0.86
**AG-O**	0.86	0.86
**AG-Fb**	0.93	0.89
**AG-P**	0.86–0.89	0.86–0.90
**AG-R**	0.88	0.85
**AG-S**	0.88	0.88
**AG-Fa**	0.91	0.91
**AG-E**	0.90–0.91	0.89
**UNR1**	0.82–0.83	0.84
**UNR2**	0.80	0.82
**AG-N**	0.67	0.66
***W. circinata***	0.66–0.67	0.64–0.66

LDDL02-1 shows most similarity to AG 6 and AG-Fb. DNBH05-1-2 shows highest pairwise sequence similarity to AG 1-IA and AG 1-IE.

**Figure 5:**
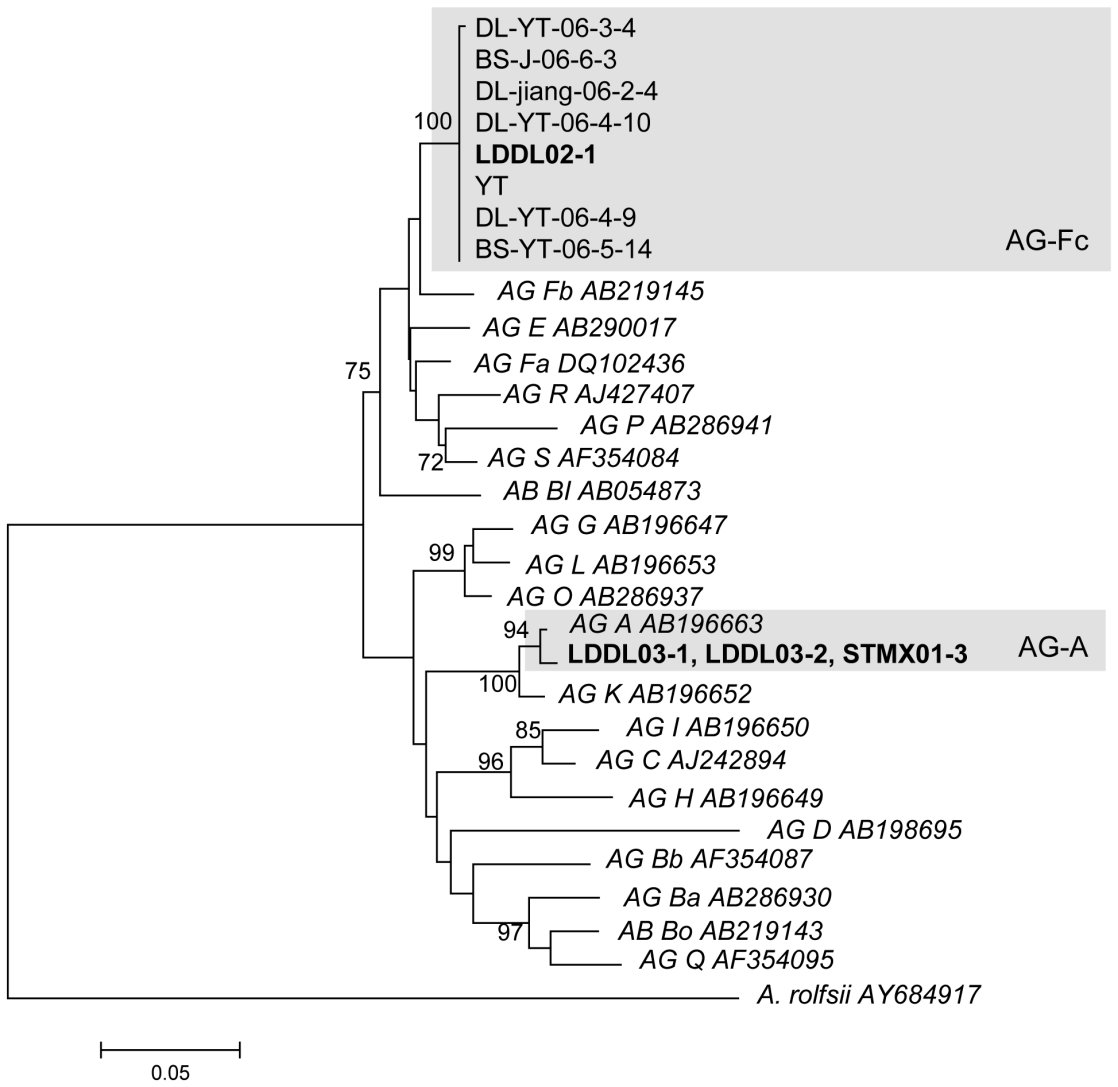
rDNA-ITS phylogeny of binucleate *Rhizoctonia* spp. sampled from *Brassica* spp. in Vietnam. Neighbour joining tree derived from the alignment of 31 binucleate *Rhizoctonia* isolates and the outgroup *Athelia rolfsii* (AY684917). Isolates in bold are the 4 isolates derived from *Brassica* spp. in Vietnam during this study. For each known binucleate *Rhizoctonia* AG, a representative isolate (in italics) from the curated database (Table S2 in File S2) is included. Bootstraps are only given for those branches with bootstrap support higher than 70. The tree was made using only isolates with unique sequences. Isolates with identical sequences were added afterwards on the same line.

From the binucleate phylogenetic tree (Figure 5), it is clear that the unknown isolate LDDL02-1 clusters together with the isolates from Yunnan province, forming a clade with high bootstrap support that is different from any known binucleate AG, with the closest related AG being AG-Fb. So far, nothing has been published about the Chinese isolates. Pairwise sequence similarity within AG-F is 90–100% [2], and the closest related AGs are AG-Fb (93%) and AG-Fa (91%). Therefore, we propose to assign these isolates as a new AG-F subclade, namely AG-Fc.

**Figure 6:**
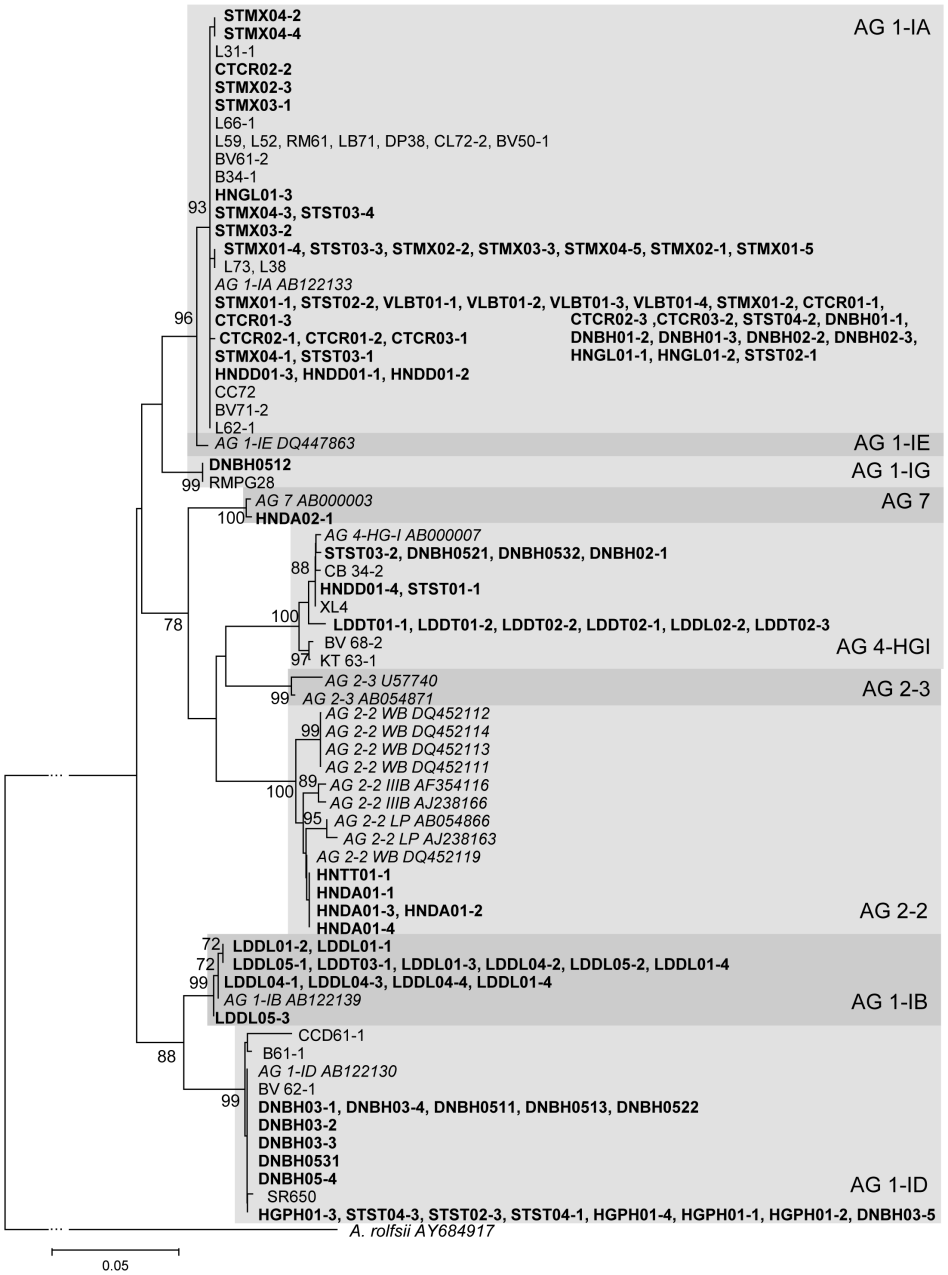
rDNA-ITS phylogeny of multinucleate *Rhizoctonia* spp. sampled from *Brassica* spp. in Vietnam. Neighbour joining tree derived from the alignment of 128 multinucleate *Rhizoctonia* isolates and the outgroup *Athelia rolfsii* (AY684917). Isolates in bold are the isolates derived from *Brassica* spp. in Vietnam during this study. For each of the multinucleate *Rhizoctonia* AG subgroups present in our sampling, representative isolates (in italics) from the curated database (Table S2 in File S2) are included. Bootstraps are only given for those branches with bootstrap support higher than 70. The tree was made using only isolates with unique sequences. Isolates with identical sequences were added afterwards on the same line. Only half of the length of the outgroup branch is shown to increase clarity.

In the multinucleate tree, all our Vietnamese isolates form clades with high bootstrap supports together with representative isolates from the curated database (Figure 6). The largest group belonged to AG 1-IA (44 isolates), followed by AG 1-ID (17 isolates), AG 1-IB (13 isolates) and AG 4-HGI (12 isolates). Five isolates belonged to AG 2-2. AG 2-2 is further divided into four groups: AG 2-2 IV, AG 2-2 LP, AG 2-2 IIIB and AG 2-2 WB. The similarity scores between our isolates and AG 2-2 IIIB and AG 2-2 LP were comparable (between 97 and 98%). Also, our isolates show 99% similarity with isolate Barranca (DQ452119), which is assigned as AG 2-2 WB by Godoy-Lutz et al. [25]. However, with other isolates from AG 2-2 WB (DQ452111-114), pairwise sequence similarity is lower (between 96 and 97%). Therefore, we decided that it is impossible at this point to assign our isolates to any of the AG 2-2 phylogenetic subgroups. One isolate belonged to AG 7 and the last isolate (DNBH05-1-2) could not be assigned to any of the known multinucleate AG groups in our reference database. The isolate showed the highest pairwise sequence similarity with AG 1-IE (94%, see Table 4). A BLASTn search on Genbank identified an isolate from chickpea in India (RMPG28, [29]) assigned to AG 2–3 to be>99% similar (1 bp substitution) to DNBH05-1-2. However, when comparing these isolates to the AG 2–3 isolates in the curated database, we found low pairwise sequence similarity (85%) (Table 4). Also in the phylogenetic tree (see Figure 6), isolate DNBH05-1-2 clusters together with isolate RMPG28, but not with the representative AG 2–3 isolates from the curated database, indicating that isolate RMPG28 is wrongly designated as AG 2–3 in Genbank. Hence, these isolates represent a new subgroup of AG 1 and we propose the name AG 1-IG.

For most of the AGs we detected in Vietnam there was very little variation in ITS sequence, except for AG 4-HGI where six variable positions were found between the isolates from Lam Dong and the isolates from the other three provinces where this AG was detected (Soc Trang, Dong Nai and Ha Noi).

The AG 1-IA isolates showed identical or nearly identical (max. 4 SNPs) sequences to AG 1-IA isolates previously isolated from rice, water spinach, water hyacinth and other crops in Vietnam (Table 3). The AG 1-ID isolates showed high similarity (identical sequence or one SNP) to isolates detected on coffee, cotton, durian [30], corn and sugar beet (Table 3) in Vietnam.

### Relationship between the AGs found and sampling locations and between AGs found and *Brassica* species

There seems to be a relationship between the AGs present and the sampling areas (Figure 1 and Table 5). Thirty three out of 44 isolates belonging to AG 1-IA were recovered from samples collected from Soc Trang, Can Tho and Vinh Long in the Mekong River delta, the main rice production region of Vietnam. On the other hand, no AG 1-IA isolates were detected in Lam Dong although the expected frequency of this group in this province was high (9.98). The observed numbers were also significantly higher than the expected numbers for AG 1-IB and AG 4-HGI in Lam Dong as well as for AG 1-ID in Hau Giang and Dong Nai.

**Table 5:** Contingency table with observed and expected frequencies of anastomosis groups (AGs) of *Rhizoctonia* spp. obtained from *Brassica* fields in different provinces of Vietnam.

Province	AG/Subset									Total/province
	Multinucleate *Rhizoctonia*							Binucleate *Rhizoctonia*		
	1-IA	1-IB	1-ID	2-2	1-IG	4-HGI	7	A	Fc	
**Ha Noi**	6 (5.90)	0 (1.74)	0 (2.28)	5 (0.67)	0 (0.13)	1 (1.61)	1 (0.13)	0 (0.40)	0 (0.13)	13
**Lam Dong**	0 (9.98)*	13 (2.95)*	0 (3.86)	0 (1.13)	0 (0.45)	6 (2.72)*	0 (0.23)	2 (0.68)	1 (0.23)	22
**Dong Nai**	5 (8.62)	0 (2.55)	10 (3.33)*	0 (0.98)	1 (0.20)	3 (2.35)	0 (0.20)	0 (0.59)	0 (0.20)	19
**Vinh Long**	4 (1.81)	0 (0.54)	0 (0.70)	0 (0.21)	0 (0.04)	0 (0.49)	0 (0.04)	0 (0.12)	0 (0.04)	4
**Can Tho**	8 (3.63)	0 (1.07)	0 (1.40)	0 (0.41)	0 (0.08)	0 (0.99)	0 (0.08)	0 (0.25)	0 (0.08)	8
**Hau Giang**	0 (1.81)	0 (0.54)	4 (0.70)*	0 (0.21)	0 (0.04)	0 (0.49)	0 (0.04)	0 (0.12)	0 (0.04)	4
**Soc Trang**	21 (12.25)*	0 (3.62)	3 (4.73)	0 (1.39)	0 (0.28)	2 (3.34)	0 (0.28)	1 (0.84)	0 (0.28)	27
**Total/AG**	44	13	17	5	1	12	1	3	1	97

Data show actual numbers of isolates collected among the different provinces. According to Fisher's exact test (p = 0.05), the AGs found are related to the sampling locations. An asterisk* indicates significant differences between observed and expected numbers. Values in parentheses represent the expected numbers.

The relationship between the occurrence of different AGs and the host plants is presented in Table 6. Significant AG-host correlations were identified in the following combinations: AG 1-IB and white cabbage, AG 1-IB and broccoli, AG 1-ID and Chinese flowering cabbage, and AG 2-2 and turnip cabbage.

**Table 6:** Contingency table with observed and expected frequencies of anastomosis groups (AGs) of *Rhizoctonia* spp. obtained from field-grown *Brassica* crops in Vietnam.

Host plant	AG/Subset^a^									Total/host plant
	Multinucleate *Rhizoctonia*							Binucleate *Rhizoctonia*		
	1-IA	1-IB	1-ID	2-2	1-IG	4-HGI	7	A	Fc	
**Mustard cabbage**	14 (9.07)	0 (2.68)	5 (3.51)	0 (1.03)	0 (0.21)	1 (2.47)	0 (0.21)	1 (0.62)	0 (0.21)	20
**White cabbage**	8 (9.53)	8 (2.81)*	0 (3.68)	0 (1.08)	0 (0.22)	3 (2.60)	0 (0.22)	2 (0.65)	0 (0.22)	21
**Chinese flowering cabbage**	9 (12.25)	0 (3.62)	12 (4.73)*	1 (1.39)	1 (0.28)	4 (3.34)	0 (0.28)	0 (0.84)	0 (0.28)	27
**Chinese cabbage**	0 (2.27)	1 (0.67)	0 (0.88)	0 (0.26)	0 (0.05)	3 (0.62)	0 (0.05)	0 (0.15)	1 (0.05)	5
**Pak choi**	10 (4.99)	0 (1.47)	0 (1.93)	0 (0.57)	0 (0.11)	0 (1.36)	0 (0.11)	1 (0.34)	0 (0.11)	11
**Broccoli**	0 (1.81)	4 (0.54)*	0 (0.70)	0 (0.21)	0 (0.04)	0 (0.49)	0 (0.04)	0 (0.12)	0 (0.04)	4
**Turnip cabbage**	3 (4.08)	0 (1.21)	0 (1.58)	4 (0.46)*	0 (0.09)	1 (1.11)	1 (0.09)	0 (0.28)	0 (0.09)	9
**Total/AG**	44	13	17	5	1	12	1	3	1	97

Data show actual numbers of isolates collected among the different *Brassica* crops. According to Fisher's exact test (p = 0.05), the AGs found are related to the crops. An asterisk* indicates significant differences between observed and expected numbers. Values in parentheses represent the expected numbers.

### Aggressiveness of *Rhizoctonia* isolates towards detached leaves

Nine *Rhizoctonia* isolates, randomly selected from each identified AG, were tested for their pathogenicity on detached leaves. Due to practical reasons, experiments with *Brassica* crops were conducted in a growth chamber specifically built for *Brassica* spp. (22°C, RH = 60%, 12 h photoperiod) and experiments with rice and water spinach were done in a growth chamber specifically built for rice (28°C, RH = 60%, 16 h photoperiod). Although the temperature and humidity in these chambers are slightly lower than those of Vietnam, they are still suitable for the growth of host plants and *Rhizoctonia* isolates involved in our study.

As shown in Figure 2, leaves of white cabbage, Chinese cabbage, pak choi, mustard cabbage, Chinese flowering cabbage, rice, and water spinach responded differently to the infection of *Rhizoctonia* spp. although each host was affected by at least four AGs. For each plant species, a wide variation in symptom severity induced by different AGs was observed and the only AG that could not cause disease on any of the plants tested was AG-A. White cabbage leaves were most severely infected by all AGs (except AG-A) with a DI varying from 3.13 to 4.00. Compared to other hosts, Chinese cabbage appeared to be most resistant to *Rhizoctonia* isolates. No disease symptoms were observed on Chinese cabbage leaves challenged with AG 1-IB, AG 1-ID, AG 2-2 and AG 7, while other AGs were weak to moderately aggressive (DI varied from 0.71 in AG-Fc to 2.63 in AG 1-IA). Inoculation of pak choi with AG 1-IB resulted in a complete decay of all leaf discs (DI = 4.00). For mustard cabbage and Chinese flowering cabbage, a strong disease pattern was obtained on leaves confronted with AG 1-IB (DI≥3.92) and AG 4-HGI (DI≥3.71). Rice leaves were very susceptible to AG 1-IA and they were severely destroyed within four days of inoculation (DI = 3.92). Disease induced by other *R. solani* isolates on rice was also significantly different from the control although the two binucleate *Rhizoctonia* isolates tested were not pathogenic on rice. For water spinach, a wide variation in aggressiveness of *R. solani* isolates was observed, resulting in DI values ranging from 0.46 to 3.54. Large lesions (DI≥3.21) developed on water spinach leaves inoculated with AG 1-IA and AG 1-ID isolates. The difference in pathogenicity of these AGs towards the different *Brassica* species was confirmed when additional isolates from AGs 1-IA, 1-IB, 1-ID, 2-2, 4-HGI and A were tested in a detached leaf assay on the same series of plants (see supplementary information, Table S3 in File S1).

### *In vitro* pathogenic potential of *Rhizoctonia* spp. isolates on seedlings of white cabbage and Chinese cabbage

The results of the detached leaf assay revealed that white cabbage leaves were remarkably more susceptible than other cabbage species, while only some AGs could attack the leaves of Chinese cabbage. Therefore, these two plant species were selected as hosts for *in vitro* and *in vivo* pathogenicity tests using the same nine *Rhizoctonia* isolates as mentioned above. Under *in vitro* conditions (Figure 3), all AGs could cause disease and symptoms were observed on both roots and leaves. Isolates of AG 1-IA, AG 1-IB, AG 2-2, AG 1-IG, AG 4-HGI and AG-Fc were most aggressive towards these hosts (for white cabbage: DI on roots ≥3.63 and DI on leaves ≥3.42; for Chinese cabbage: DI on roots ≥3.08 and DI on leaves ≥2.71). Fewer symptoms were recorded on seedlings inoculated with isolates belonging to AG 1-ID, AG 7 and AG-A. The virulence of AG 1-IA, AG 1-IB, AG 2-2 and AG 4-HGI towards white cabbage and Chinese cabbage was confirmed when *in vitro* assays were repeated with additional isolates of these AGs (Table S4 in File S1). Towards white cabbage, DI on roots varied from 2.17 to 4.00 and DI on leaves fluctuated between 2.33 and 4.00. DI on roots and leaves of Chinese cabbage ranged from 2.08 to 4.00 and from 1.92 to 4.00, respectively.

### *In vivo* pathogenic potential of *Rhizoctonia* spp. isolates on roots and hypocotyls of white cabbage and Chinese cabbage

The results displayed in Figure 4 demonstrate that severe *Rhizoctonia*-induced damage on white cabbage roots could only be seen for AG 2-2 (DI = 3.50) and AG 4-HGI (DI = 3.63). Moderate infection (DI = 2.33) was observed in response to AG-Fc. Towards Chinese cabbage, severe disease symptoms were incited by AG 4-HGI (DI = 3.08) and AG-Fc (DI = 2.96). No symptoms were detected on roots and hypocotyls of seedlings challenged with AG-A. In addition, the inoculation of isolates belonging to AG 1-IA, AG 1-IB, AG 1-ID, AG 1-IG and AG 7 did not result in the formation of large lesions on roots of white cabbage and Chinese cabbage seedlings (DI≤1.57). Data obtained from the tests conducted with additional *Rhizoctonia* isolates belonging to AGs 1-IA, 1-IB, 1-ID, 2-2, 4-HGI and AG-A are presented in Table S5 in File S1. These data confirm the high aggressiveness of AG 2-2 on white cabbage (DI = 4.00) and that of AG 4-HGI on both white cabbage and Chinese cabbage (DI≥3.50).

## Discussion

*Rhizoctonia* is an important fungal ‘form genus’ occurring worldwide and including many important plant pathogenic strains as well as mycorrhizal fungi and hypovirulent or avirulent strains among which there are strains that are capable to protect plants against pathogenic *Rhizoctonia* and other pathogens as well as increase plant growth. Plants can be infected by different *Rhizoctonia* AGs from the time of sowing resulting in the development of both foliar and root diseases. This is the first time *Rhizoctonia* species that attack field-grown *Brassica* crops in Vietnam were isolated and characterized. Ninety seven isolates of *Rhizoctonia* were recovered from symptomatic plant tissues of seven brassicaceous hosts (mustard cabbage, white cabbage, Chinese flowering cabbage, pak choi, turnip cabbage, Chinese cabbage and broccoli). Of all the isolates collected, 4% were binucleate *Rhizoctonia* and 96% were multinucleate *Rhizoctonia*. Molecular characterization by sequencing of the rDNA-ITS region showed that the binucleate isolates found belonged to AG-A (3 isolates) and an unknown AG introduced here as AG-Fc (1 isolate). Comparison to a curated sequence database of all known *Rhizoctonia* multinucleate, binucleate and uninucleate AGs did not reveal high homology of this isolate to a known AG. Pairwise sequence similarities to all known multinucleate, binucleate and uninucleate *Rhizoctonia* AGs showed highest similarity to AG-Fb, but also to AG 6, which is a multinucleate AG. Close relationships of some binucleate groups with multinucleate groups has been previously noted by Sharon et al. [2] who did a combined ITS sequence analysis of multinucleate, binucleate and uninucleate groups. These authors stated that the clustering of binucleate and uninucleate groups close to certain multinucleate clusters may indicate a possible evolutionary bridge between multinucleate and binucleate groups and our results support this hypothesis.

We conducted pathogenicity assays on detached leaves, *in vitro* seedlings and plants grown *in vivo*. In general, pathogenicity was highest on roots and leaves under *in vitro* conditions, which is probably due to the high humidity and the young age of the plants in this system. It should be mentioned that we only checked *in vivo* pathogenicity towards roots and hypocotyls, but it should be borne in mind that some of our isolates are mainly leaf pathogens.

Although AG-A isolates were obtained from symptomatic plants, they were unable to induce disease on detached leaves or on seedlings *in vivo*, and were only slightly pathogenic on cabbage seedlings under *in vitro* conditions. This low virulence may be due to the differences in humidity between our experimental conditions and the field situation in Vietnam. Alternatively, AG-A isolates might be avirulent, but since they grow very fast *in vitro*, they may have masked the presence of slower growing, virulent AGs. In contrast to AG-A, AG-Fc appeared to be moderately to highly virulent in all experiments. This also concurs with previous studies that some binucleate *Rhizoctonia* species are highly virulent [31]–[33], whereas others are weakly virulent or avirulent [34]–[36].

Among the multinucleate AGs, AG 1-IA was the dominant group, followed by AG 1-ID, AG 1-IB, AG 4-HGI, AG 2-2, AG 7 and an unknown AG that we introduced here as AG 1-IG. The occurrence of AG 1-IB and AG 4 on *Brassica* spp. is well known from previous studies. According to Pannecoucque et al. [22], AG 1-IB is one of the causal agents of wirestem in Belgian cauliflower fields. AG 1-IB isolates are also highly virulent on lettuce in Belgium [37]. In our study, AG 1-IB isolates were only recovered from Lam Dong, a province in the cool Central Highlands of Vietnam and the main lettuce production region of Vietnam, suggesting that the presence of this AG is associated with cool climates. The presence of AG 4 has also been reported on *Brassica oleracea* [21] and *B. rapa* subsp. *chinensis* [38] in China, *B. oleracea* in the UK [23], *B. napus* L. and *B. campestris* L. in Canada [39], and *B. oleracea* [18] and *B. napus* L. in the US [40]. AG 4-HGI occurred in most regions sampled. This AG has a wide host range due to its ability to adapt to temperature variation and cropping patterns [41]. AG 4 isolates are able to induce disease on all plant parts and in our pathogenicity trials, the highest disease ratings on cabbage in all bioassays were shown for the AG 4-HGI isolates. For white cabbage, severe disease symptoms were observed on seedlings inoculated with AG 2-2 and AG 4-HGI isolates *in vivo* or on detached leaves challenged with isolates of all AGs. Although *R. solani* AG 2-1 is considered the most dominant and damaging anastomosis group attacking *Brassica* spp. [22], [23], [42], isolates belonging to this group were not detected in our survey.

**Figure 7:**
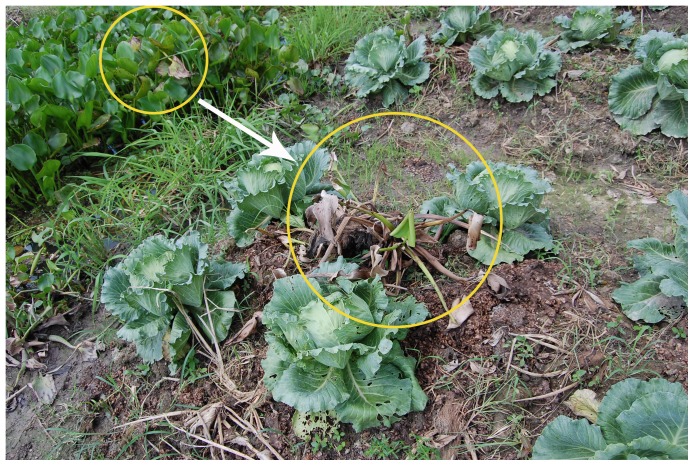
*Rhizoctonia*-infected water hyacinth is introduced as cover material to a white cabbage field in Vietnam. *Rhizoctonia*-infected water hyacinth is taken from a nearby water ditch and used as cover material on the white cabbage field. Via this common practice, Vietnamese farmers unintentionally introduce the *Rhizoctonia* fungus to their crops.

The predominance of *R. solani* isolates of AG 1-IA and also the presence of isolates belonging to AG 1-ID in our collection were not anticipated because these AGs have not been described on *Brassica* crops before. The presence of unusual AGs in our sampling is probably due to three aspects: (i) alternative hosts of *Rhizoctonia* present in the sampling locations; (ii) poor cultural practices and (iii) high temperature. AG 1-IA isolates were mainly recovered from samples collected in provinces of the Mekong delta including Vinh Long, Can Tho and Soc Trang. This is a low-lying coastal region of Vietnam, characterized by high temperature and humidity and prone to flooding every rainy season. Due to water availability and soil type, the agricultural production in this area is dominated by rice [43], [44]. As previously reported, sheath blight, caused by *R. solani* AG 1-IA, is a major disease of rice cultivated in intensive production systems [45]–[47]. With the ability to float and to survive in water [48], sclerotia of AG 1-IA isolates may easily spread from the rice paddy fields to vegetable fields through irrigation or flood water. The recovery of AG 1-IA in the hot region located in the South of Vietnam is consistent with the findings of Harikrishnan and Yang [41] that temperature can influence growth rate, sclerotia production of *Rhizoctonia* spp. and the distribution of *Rhizoctonia* isolates belonging to different anastomosis groups. AG 1 is a high temperature group [8] and its vegetative growth as well as sclerotia production and survival are inhibited at low temperatures. The occurrence of AG 1-IA on *Brassica* crops is probably even increased due to farmers' lack of knowledge about crop protection. AG 1 (not specific) has been considered as one of the causal agents of foliar diseases on water hyacinth (*Eichhornia crassipes*) [49], water lettuce (*Pistia stratioites*) and anchoring hyacinth (*E. azurea*) [50]. In this study, water spinach (*Ipomoea aquatic*) was found to be susceptible to the AG 1-IA isolates that we collected from *Brassica* spp. Vietnamese farmers commonly use these aquatic plants as cover materials in vegetable production and use irrigation water from sources where these plants are present, thus bringing the fungus from these alternative hosts to *Brassica* spp. (Figure 7). Isolates of AG 1-IA appeared to be very pathogenic towards leaves of cabbages but they could not induce severe disease on roots, especially under *in vivo* conditions. This finding is in agreement with results reported previously by Yang and Li [51] that AG 1-IA isolates have a tendency to attack aerial parts of plants. These data also support our hypothesis about the spread of AG 1-IA isolates from rice and water spinach to vegetables. In other words, rice and water spinach that are infected by *R. solani* could be an important source of inoculum that may contribute to the disease caused by AG 1-IA on *Brassica* spp.

In our assays AG 1-ID was mainly pathogenic on leaves of white cabbage. *Rhizoctonia* AG 1-ID was previously found to be pathogenic on durian [30] and coffee [52]. Interestingly, ten out of 17 AG 1-ID isolates were collected from Dong Nai, a province where durian, coffee and cotton are widely cultivated [53], again suggesting a correlation between cropping patterns and *Rhizoctonia* distribution in Vietnam.

Collectively, it seems that the distribution of *Rhizoctonia* AGs in Vietnam is correlated with the cropping patterns and climatic conditions. However, it is difficult to draw a strong conclusion about the influence of cropping patterns and climatic conditions on the occurrence of *Rhizoctonia* spp. in Vietnam because the sampling regime for *R. solani* isolates comprises two variable parameters, namely the plant species infected by *R. solani* and the sampling site. Due to the variation in soil and climatic conditions, different *Brassica* species are grown in different geographic regions and there is a possibility that particular plant species might be more susceptible to infections by specific *R. solani* AGs than others. Therefore, another field survey with a systematic sampling regime needs to be conducted to confirm our hypothesis.

Our research also points towards the need to have good extension programs to improve the farmers' knowledge about crop protection. Additionally, knowing which AGs are responsible for *Rhizoctonia* diseases on *Brassica* spp. in Vietnam is an essential prerequisite for developing successful disease management strategies in this country.

## Supporting Information

File S1 Contains the following files: Table S1. GPS co-ordinates of the wards in each province of Vietnam where *Rhizoctonia*-infected *Brassica* crops were sampled. Table S3. Aggressiveness of *Rhizoctonia* isolates towards white cabbage, Chinese cabbage, pak choi, mustard cabbage, Chinese flower cabbage, rice and water spinach in detached leaf bio-assays. Leaves were scored using a scale ranging from 0 (no disease symptoms) to 4 (lesions covered more than 75% of leaf surface or dead leaf). For rapid visual evaluation of the data, a coloring scale with green (0<DI≤1), yellow (1<DI≤2), orange (2<DI≤3) and red (3<DI≤4) was used. The test was done once with 12 leaves or leaf pieces per treatment. All data were statistically analyzed and within columns, disease severities followed by the same letter are not significantly different. Table S4. Aggressiveness of *Rhizoctonia* isolates towards roots and leaves of white cabbage and Chinese cabbage seedlings in *in vitro* bio-assays. Disease severity on roots or leaves was assessed on a scale ranging from 0 (no symptoms) to 4 (lesions covering more than 75% of root, hypocotyl or leaf surface or dead plant). For rapid visual evaluation of the data, a coloring scale with green (0<DI≤1), yellow (1<DI≤2), orange (2<DI≤3) and red (3<DI≤4) was used. Experiment was conducted once with 12 seedlings maintained in two square Petri plates for one treatment. All data were statistically analyzed and within columns, disease severities followed by the same letter are not significantly different. Table S5. Aggressiveness of *Rhizoctonia* isolates towards roots of white cabbage and Chinese cabbage seedlings in in planta experiment. Disease severity on roots was assessed on a scale ranging from 0 (no symptoms) to 4 (seedling dead). For rapid visual evaluation of the data, a coloring scale with green (0<DI≤1), yellow (1<DI≤2), orange (2<DI≤3) and red (3<DI≤4) was used. Experiment was performed once. Each treatment consisted of 12 seedlings cultivated in two plastic boxes. Data were statistically analyzed and within columns, disease severities followed by the same letter are not significantly different.
(DOC)

File S2 **Table S2.** Curated database of sequences containing representative rDNA-ITS sequences of all known uninucleate, binucleate and multinucleate *Rhizoctonia* AG and subgroups.
(XLSX)
