# Supplementary material for: Cropping Systems and Cultural Practices Determine the Rhizoctonia Anastomosis Groups Associated with Brassica spp. in Vietnam
Source: PLoS One. 2014 Nov 5;9(11):e111750. doi: 10.1371/journal.pone.0111750 (PMC4221111; doi:10.1371/journal.pone.0111750)
Supplement: File S1 — Contains the following files: Table S1. GPS co-ordinates of the wards in each province of Vietnam where Rhizoctonia-infected Brassica crops were sampled. Table S3. Aggressiveness of Rhizoctonia isolates towards white cabbage, Chinese cabbage, pak choi, mustard cabbage, Chinese flower cabbage, rice and water spinach in detached leaf bio-assays. Leaves were scored using a scale ranging from 0 (no disease symptoms) to 4 (lesions covered more than 75% of leaf surface or dead leaf). For rapid visual evaluation of the data, a coloring scale with green (0<DI≤1), yellow (1<DI≤2), orange (2<DI≤3) and red (3<DI≤4) was used. The test was done once with 12 leaves or leaf pieces per treatment. All data were statistically analyzed and within columns, disease severities followed by the same letter are not significantly different. Table S4. Aggressiveness of Rhizoctonia isolates towards roots and leaves of white cabbage and Chinese cabbage seedlings in in vitro bio-assays. Disease severity on roots or leaves was assessed on a scale ranging from 0 (no symptoms) to 4 (lesions covering more than 75% of root, hypocotyl or leaf surface or dead plant). For rapid visual evaluation of the data, a coloring scale with green (0<DI≤1), yellow (1<DI≤2), orange (2<DI≤3) and red (3<DI≤4) was used. Experiment was conducted once with 12 seedlings maintained in two square Petri plates for one treatment. All data were statistically analyzed and within columns, disease severities followed by the same letter are not significantly different. Table S5. Aggressiveness of Rhizoctonia isolates towards roots of white cabbage and Chinese cabbage seedlings in in planta experiment. Disease severity on roots was assessed on a scale ranging from 0 (no symptoms) to 4 (seedling dead). For rapid visual evaluation of the data, a coloring scale with green (0<DI≤1), yellow (1<DI≤2), orange (2<DI≤3) and red (3<DI≤4) was used. Experiment was performed once. Each treatment consisted of 12 seedlings cultivated in two plast [file pone.0111750.s001.doc]

**Supporting Information**

**Table S1:** GPS co-ordinates of the wards in each province of Vietnam where *Rhizoctonia*-infected *Brassica* crops were sampled.

| **Province** | **District / City** | **Ward** | **Latitude** | **Longitude** |
| --- | --- | --- | --- | --- |
| Ha Noi | Gia Lam | Dong Du | 21° 0'19.04"N | 105°55'4.36"E |
|  | Thanh Tri | Ngoc Hoi | 20°55'36.59"N | 105°50'36.69"E |
|  | Dong Anh | Nam Hong | 21°10'4.17"N | 105°47'32.86"E |
|  |  | Van Noi | 21° 9'11.05"N | 105°48'54.83"E |
| Lam Dong | Da Lat city | Ward 6 | 11°58'3.28"N | 108°26'1.98"E |
|  |  | Ward 8 | 11°59'38.29"N | 108°27'30.02"E |
|  | Duc Trong | Ninh Loan | 11°34'25.74"N | 108°20'42.95"E |
|  |  | Phu Hoi | 11°40'39.69"N | 108°21'30.33"E |
| Dong Nai | Bien Hoa | Tan Bien | 10°58'59.62"N | 106°53'56.66"E |
| Vinh Long | Binh Tan | Tan Quoi | 10° 6'8.14"N | 105°45'36.02"E |
| Can Tho | Cai Rang | Hung Phu | 10° 2'9.99"N | 105°47'45.59"E |
|  |  | Thuong Thanh | 9°59'21.92"N | 105°45'24.03"E |
| Hau Giang | Phung Hiep | Long Thanh | 9°53'16.55"N | 105°44'36.64"E |
| Soc Trang | Soc Trang | Ward 3 | 9°35'22.39"N | 105°58'36.91"E |
|  | My Xuyen | Dai Tam | 9°33'3.54"N | 105°55'10.88"E |

**Table S3:** Aggressiveness of *Rhizoctonia* isolates towards white cabbage, Chinese cabbage, pak choi, mustard cabbage, Chinese flower cabbage, rice and water spinach in detached leaf bio-assays.

| **AG / Subgroup** | **Isolate** | **Disease Index** | | | | | | |
| --- | --- | --- | --- | --- | --- | --- | --- | --- |
|  |  | **White cabbage** | **Chinese cabbage** | **Pak choi** | **Mustard cabbage** | **Chinese flowering cabbage** | **Rice** | **Water spinach** |
|  | Control | 0.00 a | 0.00 a | 0.00 a | 0.00 a | 0.00 a | 0.00 a | 0.00 a |
| 1-IA | VLBT01-4 | 4.00 c | 1.75 b | 2.50 de | 1.33 c | 1.92 cd | 4.00 d | 3.75 e |
|  | CTCR02-3 | 3.83 bc | 2.17 bc | 1.67 cd | 1.00 c | 2.67 de | 3.75 d | 3.33 e |
|  | STST03-3 | 4.00 c | 2.75 c | 1.67 cd | 1.08 c | 2.25 d | 3.75 d | 3.50 e |
|  | STMX02-1 | 4.00 c | 2.67 c | 1.50 c | 1.17 c | 2.08 cd | 4.00 d | 3.25 de |
| 1-IB | LDDL04-1 | 4.00 c | 0.00 a | 3.83 f | 4.00 f | 3.75 fg | 1.08 b | 3.00 de |
|  | LDDL05-2 | 4.00 c | 0.50 a | 4.00 f | 3.75 ef | 3.92 g | 1.00 b | 3.00 de |
| 1-ID | HGPH01-4 | 4.00 c | 0.00 a | 0.50 b | 1.67 cd | 1.25 bc | 1.75 c | 2.67 cd |
|  | STST02-3 | 4.00 c | 0.00 a | 0.58 b | 2.00 de | 1.75 cd | 1.42 bc | 2.92 de |
|  | DNBH05-1-1 | 3.17 b | 0.00 a | 0.42 b | 1.83 d | 1.67 cd | 1.67 c | 2.75 cde |
| 2-2 | HNTT01-1 | 4.00 c | 0.00 a | 0.50 b | 0.00 a | 0.00 a | 1.08 b | 1.92 bc |
| 4-HGI | DNBH05-3-2 | 3.00 b | 2.92 c | 2.92 e | 3.75 ef | 3.17 ef | 1.17 b | 2.17 c |
|  | LDDT02-3 | 4.00 c | 2.75 c | 3.42 ef | 3.67 ef | 3.33 f | 1.42 bc | 1.50 b |
| A | LDDL03-1 | 0.00 a | 0.00 a | 0.00 a | 0.00 a | 0.00 a | 0.00 a | 0.00 a |
|  | LDDL03-2 | 0.00 a | 0.00 a | 0.00 a | 0.00 a | 0.00 a | 0.00 a | 0.00 a |

Leaves were scored using a scale ranging from 0 (no disease symptoms) to 4 (lesions covered more than 75% of leaf surface or dead leaf). For rapid visual evaluation of the data, a coloring scale with green (0 < DI ≤ 1), yellow (1 < DI ≤ 2), orange (2 < DI ≤ 3) and red (3 < DI ≤ 4) was used. The test was done once with 12 leaves or leaf pieces per treatment. All data were statistically analyzed and within columns, disease severities followed by the same letter are not significantly different.

**Table S4:** Aggressiveness of *Rhizoctonia* isolates towards roots and leaves of white cabbage and Chinese cabbage seedlings in *in vitro* bio-assays.

| **AG/Subgroup** | **Isolate** | **Disease Index** | | | |
| --- | --- | --- | --- | --- | --- |
|  |  | **Root** | | **Leaf** | |
|  |  | **White cabbage** | **Chinese cabbage** | **White cabbage** | **Chinese cabbage** |
|  | Control | 0.00 a | 0.00 a | 0.00 a | 0.00 a |
| 1-IA | VLBT01-4 | 3.67 cd | 2.67 bc | 2.75 def | 2.17 cd |
|  | CTCR02-3 | 2.17 b | 2.42 bc | 2.33 cde | 2.75 d |
|  | STST03-3 | 2.58 bc | 2.08 bc | 2.92 def | 2.33 cd |
|  | STMX02-1 | 2.42 bc | 3.25 cd | 3.08 ef | 1.92 bcd |
| 1-IB | LDDL04-1 | 4.00 d | 3.58 cd | 4.00 g | 2.92 de |
|  | LDDL05-2 | 3.17 c | 3.83 cd | 3.33 f | 3.17 def |
| 1-ID | HGPH01-4 | 2.25 bc | 1.83 b | 1.25 b | 1.17 b |
|  | STST02-3 | 3.33 c | 1.92 b | 3.33 f | 1.00 b |
|  | DNBH05-1-1 | 1.92 b | 1.50 b | 2.17 bcde | 1.42 bc |
| 2-2 | HNTT01-1 | 4.00 d | 3.83 cd | 4.00 g | 2.42 cd |
| 4-HGI | DNBH05-3-2 | 4.00 d | 4.00 d | 4.00 g | 4.00 f |
|  | LDDT02-3 | 4.00 d | 3.92 d | 4.00 g | 3.83 ef |
| A | LDDL03-1 | 1.67 b | 1.75 b | 1.50 bc | 1.42 bc |
|  | LDDL03-2 | 2.08 b | 1.75 b | 1.67 bcd | 1.45 bc |

Disease severity on roots or leaves was assessed on a scale ranging from 0 (no symptoms) to 4 (lesions covering more than 75% of root, hypocotyl or leaf surface or dead plant). For rapid visual evaluation of the data, a coloring scale with green (0 < DI ≤ 1), yellow (1 < DI ≤ 2), orange (2 < DI ≤ 3) and red (3 < DI ≤ 4) was used. Experiment was conducted once with 12 seedlings maintained in two square Petri plates for one treatment. All data were statistically analyzed and within columns, disease severities followed by the same letter are not significantly different.

**Table S5:** Aggressiveness of *Rhizoctonia* isolates towards roots of white cabbage and Chinese cabbage seedlings in *in planta* experiment.

| **AG/Subgroup** | **Isolate** | **Disease Index** | |
| --- | --- | --- | --- |
|  |  | **White cabbage** | **Chinese cabbage** |
|  | Control | 0. 00 a | 0.00 a |
| 1-IA | VLBT01-4 | 0.25 ab | 0.42 b |
|  | CTCR02-3 | 0.27 ab | 0.58 b |
|  | STST03-3 | 0.58 bc | 0.70 b |
|  | STMX02-1 | 0.30 ab | 0.17 ab |
| 1-IB | LDDL04-1 | 0.92 bc | 1.00 bc |
|  | LDDL05-2 | 1.36 c | 0.82 b |
| 1-ID | HGPH01-4 | 0.56 b | 1.00 bc |
|  | STST02-3 | 0.25 ab | 0.42 b |
|  | DNBH05-1-1 | 0.17 ab | 0.25 ab |
| 2-2 | HNTT01-1 | 4.00 d | 1.50 c |
| 4-HGI | DNBH05-3-2 | 4.00 d | 3.67 d |
|  | LDDT02-3 | 3.50 d | 3.58 d |
| A | LDDL03-1 | 0.00 a | 0.00 a |
|  | LDDL03-2 | 0.00 a | 0.00 a |

Disease severity on roots was assessed on a scale ranging from 0 (no symptoms) to 4 (seedling dead). For rapid visual evaluation of the data, a coloring scale with green (0 < DI ≤ 1), yellow (1 < DI ≤ 2), orange (2 < DI ≤ 3) and red (3 < DI ≤ 4) was used. Experiment was performed once. Each treatment consisted of 12 seedlings cultivated in two plastic boxes. Data were statistically analyzed and within columns, disease severities followed by the same letter are not significantly different.
